# Determination of Trace Metal Levels in the General Population of Korea

**DOI:** 10.3390/ijerph14070702

**Published:** 2017-06-29

**Authors:** Hyun-Jun Kim, Hwan-Sub Lim, Kyoung-Ryul Lee, Mi-Hyun Choi, Nam Mi Kang, Chang Hoon Lee, Eun-Jung Oh, Hyun-Kyung Park

**Affiliations:** 1 Departments of Obstetrics and Gynecology, Konkuk University School of Medicine, Chungju 27376, Korea; icarus@kku.ac.kr; 2 Department of Laboratory Medicine, Seoul Clinical Laboratories, Yongin 16954, Korea; jlimhs@scllab.co.kr (H.-S.L.); dkrlee@scllab.co.kr (K.-R.L.); 3 Department of Biomedical Engineering, Research Institute of Biomedical Engineering, College of Science & Technology, Konkuk University, Chungju 27478, Korea; mhchoi0311@gmail.com; 4 College of Nursing, Konkuk University, Chungju 27478, Korea; nmkang03@kku.ac.kr; 5 Department of Laboratory Medicine, Konkuk University School of Medicine, Chungju 27376, Korea; chlee@kku.ac.kr; 6 Department of Family Medicine, Konkuk University School of Medicine, Chungju 27376, Korea; oej98@hanmail.net

**Keywords:** trace metal, inductively coupled plasma-mass spectrometry, Korea

## Abstract

The purpose of this study was to determine the levels of trace metals in the blood of the general Korean population. A total of 258 healthy individuals, according to their regular medical check-ups, (119 males and 139 females, age ranging from 12 to 78 years old) were enrolled from December 2014 to December 2016. Levels of 10 trace elements were determined using inductively coupled plasma mass spectrometry (ICP-MS). The geometric mean (GM) levels for lead, arsenic, cesium, mercury, aluminum, cadmium, copper, manganese, selenium, and zinc were 15.97 μg/L, 7.19 μg/L, 2.39 μg/L, 3.41 μg/L, 10.57 μg/L, 0.78 μg/L, 979.8 μg/L, 11.06 μg/L, 111.37 μg/L, and 872.7 μg/L, respectively. There were significant gender-related differences in the levels of several metals; male individuals had higher Pb, As, Cs, Hg, and Se than females, while females had higher Cd, Cu, and Mn than males. We noticed remarkably high blood levels of Hg, As and Al in the Korean population. The element concentrations reported represent a new contribution to the knowledge of the blood chemistry for the Korea population. The data can be used to assess the clinical health of this population.

## 1. Introduction

With the advancement of industry, there is an increasing demand for the measurement of harmful substances in the human body due to environmental pollution. Environmental pollutants such as heavy metals de-regulate the immune system, cause various adverse physiological functions and increase the prevalence and incidence of various diseases including cancer [1].

Daily exposure to contaminated food/water results in metal accumulation in the human body. Large-scale studies have been conducted in several countries to establish reference values for trace metal concentrations, which are the basis for further exposure assessment and other toxicity studies [2,3]. However, similar data on the Korean population are still lacking. In order to evaluate the degree of accumulation of heavy metals, national studies should be conducted for establishing accurate analytical techniques, selecting reference values, and establishing appropriate acceptance criteria. Investigation of trace metal concentrations through biomonitoring is effective for evaluating specific exposure profiles. In this study, we used Inductively Coupled Plasma Mass Spectrometry (ICP-MS), which has a main advantage of the simultaneous determination of the analytes. ICP-MS not only provides better performance, but also more consistent results in a variety of matrices such as serum, plasma, whole blood or urine [4,5]. The purpose of this study was to obtain data on trace metal and concentrations of a general healthy Korean population through simultaneous measurements of Lead (Pb), Arsenic (As), Cesium (Cs), Mercury (Hg), Aluminum (Al), Cadmium (Cd), Copper (Cu), Manganese (Mn), Selenium (Se) and Zinc (Zn) in the blood. We selected these ten metals because they were considered important in terms of individuals’ health and also they are used in health checks.

## 2. Materials and Methods

### 2.1. Sample Collection

Blood was obtained using a single-use stainless steel injection needle and was first used for general chemistry and blood testing. Blood used for the measurement of trace metals was collected in a BD exclusive container (BD vacutainer ™ glass sterile tube, Becton Dickinson Co., Franklin Lakes, NJ, USA). Containers with sodium heparin (catalog # 369735) were used for whole blood collection and containers without anticoagulant (catalog # 369737) were used for serum. Blood was collected using stainless steel needles which we do not believe influenced our results; these needles are commonly used for trace metal testing in clinical settings.

Seoul Clinical Laboratories (SCL)—a Reference Laboratory that provides nationwide services—collected specimens from individuals ≥12 years old and examined the profile of 10 trace metals. Individuals that had a disease, a medical history of a disease, or abnormal results in general chemistry and blood tests were excluded from the study. Collection of samples from all participants was performed in the morning fasting state and individuals were not asked to follow a special diet prior to the tests. As previously mentioned, the participants in this study were considered “general population” because they were not selected based on heavy metal exposure level or residence area. This study was approved by the Institutional Review Boards in Seoul Clinical Laboratories. Informed consent was obtained from all individual participants included in the study. As individuals under the age of consent are participants in the study, informed consent was obtained from their parents.

### 2.2. Determination of Trace Metals in the Serum and Whole Blood

Appropriate samples such as whole blood or serum were collected: Hg, Pb, Cd, Al, As, Cs and Mn were measured in the whole blood and Cu, Zn, and Se were measured in the serum.

Serum was separated immediately after 30 min of clotting time and blood and serum were kept frozen until analysis. Distilled type I (18.2 MΩ·cm) water was used throughout the handling and analysis of the samples, plastic containers were used, and samples were regularly checked for contamination. All plastic ware was cleaned, except for a disposal type with 50% nitric acid solution and distilled water before use.

Whole blood specimens were diluted 10-fold with sterile distilled water, 100 μL of 1% HNO_3_ solution (for protein removal) and 10 μg/L Scandium as an internal standard. The supernatant was taken after centrifugation at 3000 rpm for 15 min. Serum specimens were diluted 10-fold with sterile distilled water, 200 μL of 1% HNO_3_ solution and 10 μg/L Scandium as an internal standard. The supernatant was then centrifuged at 3000 rpm for 15 min.

The trace elements were analyzed with a quadrupole inductively coupled plasma-mass spectrometer equipped with a concentric glass nebulizer and a cyclonic spray chamber. Serum and whole blood trace element concentrations were determined by ICP-MS (7700× ICP-MS system, Agilent Technologies^®^, Santa Clara, CA, USA). Two isotopes of each element were measured to ensure accurate results. National Institute of Standards and Technology (NIST)-traceable 10 mg/L and 1000 mg/L elemental standards were used for preparation of multielement calibration standards. Standards passed the calibration cutoff if their back-calculated concentrations were within ±15% of the nominal concentrations. Both intra- and inter-assay imprecision were <10% of the precision coefficient of variation. The accuracy of trace elements measures was assured using the Proficiency Testing/Quality Management program of the Unites States College of American Pathologists (CAP) survey.

The limit of detection (LOD) were 0.09 μg/L for Pb, 0.14 μg/L for As, 0.16 μg/L for Cs, 0.11 μg/L for Hg, 0.38 μg/L for Al, 0.015 μg/L for Cd, 14.52 μg/L for Cu, 0.14 μg/L for Mn, 3.04 μg/L for Se and 24.93 μg/L for Zn. The limit of quantitation (LOQ) were 0.29 μg/L for Pb, 0.49 μg/L for As, 0.53 μg/L for Cs, 0.37 μg/L for Hg, 1.27 μg/L for Al, 0.05 μg/L for Cd, 43.99 μg/L for Cu, 0.49 μg/L for Mn, 9.21 μg/L for Se and 75.54 μg/L for Zn.

### 2.3. Statistical Analysis

All statistical analyses were performed using SPSS version 9.0 software (SPSS Inc., Chicago, IL, USA). The geometrical mean and standard deviation were calculated, as well as the values corresponding to 2.5, 50, 95, and 97.5 percentiles, for the specific blood trace metal concentration. In addition, histograms were used to observe the overall distribution pattern and the normal distribution was confirmed through the Goodness-of-fit test provided in the SPSS program. In addition, a non-parametric statistical method (Mann—Whitney U test) was used to analyze the effect of gender on trace metal concentrations. In order to investigate the effect of age, we compared the age group of 12–49 years with the 50–78 years age group, considering general age of socioeconomic activity. *p* values lower than 0.05 were considered as significant differences between groups.

## 3. Results

### 3.1. Characteristics of the Study Participants

The results of trace metal measurements were analyzed for a total of 258 healthy individuals who met the selection criteria set by the researchers. The characteristics of the participants, including gender and age, are presented in Table 1.

### 3.2. Serum and Whole Blood Concentrations of Trace Metals

The concentrations of the analytes in blood samples from 258 healthy individuals are presented in Table 2.

Overall, metal values did not follow a normal distribution as determined by D’ Agostino and Pearson normality test; proposed reference values for each metal analyzed are presented as percentiles and geometric means with their respective 95% upper and lower confidence intervals (Table 2).

### 3.3. Correlation between Metal Concentrations and Age or Gender

The concentrations of Pb, As, Cd and Mn in the blood were significantly different between the young and old age groups. All components except Mn were higher in the old age group (Table 3). Concentrations of Pb, As, Cd and Hg in the blood showed a positive correlation with age.

There were statistically significant differences in Pb, As, Cs, Hg, Cd, Cu, Mn, and Se concentrations depending on the gender (Table 4). Seven trace metals except Cu, Cd and Mn were higher in male individuals (Table 4). The serum Cu concentration was higher in women than in men (925 μg/L ± 170 μg/L in men, 1067 μg/L ± 243 μg/L in women). Cd and Mn concentrations in the blood were also higher in women than in men.

### 3.4. Correlation between Two Metal Concentrations

Individual correlation analysis (*n* = 258) resulted in several pairs of metals with statistically significant correlations between their concentrations (*p* < 0.05). Interestingly, we observed a correlation between Hg concentration and As (r = 0.580, *p* < 0.01), Pb (r = 0.390, *p* < 0.01), Se (r = 0.350, *p* < 0.01) and Cs (r = 0.301, *p* < 0.01) concentrations. The correlation coefficient of As and Cd (r = 0.392, *p* < 0.01) and of As and Pb (r = 0.346, *p* < 0.01) was also observed. In addition, Se and Cs (r = 0.330, *p* < 0.01) were observed.

## 4. Discussion

It is important to establish background concentrations for trace metals in the general population towards a national effort to promote public health. Al, Cd, Hg, As, Cs and Pb are classified as nonessential or toxic trace elements in this study. In contrast, Mn, Cu, Zn, and Se are classified as essential trace elements that are important for human metabolism even though their excessive accumulation in the body can be harmful.

The main sources of Pb exposure are food, water and airborne particulate matter (smoke included) [6]. As expected, reduced use of leaded gasoline and tightened control of industrial Pb emissions in industrialized countries over the last decades—resulted in a general decrease in blood Pb concentrations [6,7]. A similar reduction of blood Pb levels for the Korean population was observed; the GM of the blood Pb levels for male adults has decreased from 39.8 μg/L in 1999 [8], to 26.1 μg/L in 2005 [9], to 22.7 μg/L in 2008 [10]. The current study revealed a GM of 15.97 μg/L for blood Pb in this Korean population, which is lower than that of Chinese (34.9 μg/L), Italian (33.4 μg/L), Spanish (46.7 μg/L), and Brazilian (65.4 μg/L) populations.

In this study, blood Pb concentration was significantly higher in male (21.54 μg/L) than female participants (15.07 μg/L) (*p* < 0.01). A previous study of Korean population reported a blood Pb concentration of 22.7 μg/L for male and 16.1 μg/L for female individuals in 2012, and in both of the previous studies, the average concentration of males was higher than that of females [10,11]. It has been reported that Pb accumulation in organ tissues (hair, liver, blood, etc.) is higher in male than in female individuals, and these results are corroborated in our study. These differences in blood Pb levels between male and female individuals could be associated with differences in lifestyle and occupation-related Pb exposure [12].

Blood Pb concentration in adults has been shown to increase as a function of age [13,14]. In accordance, our data showed higher blood Pb concentration for the age group of 50–78 years (19.35 μg/L) than for the group of 12–49 years (16.95 μg/L) (*p* = 0.011).

In this study, As level was 7.19 μg/L. These levels are higher than those reported for people in France (1.67 μg/L) [15], Brazil (1.10 μg/L) [5], or China (2.33 μg/L) [16]. Human exposure to inorganic As occurs predominately through the diet and drinking water. Dietary exposure to As is the main source when As levels in the drinking water are low. Exposure to organic forms of As primarily occurs via seafood consumption. Seaweed is consumed alone or as an ingredient of soup stocks and processed foods in Korea. Seaweed consumption is also high in various other Asian countries, including Japan and China. Therefore, it is anticipated that urinary As levels will be higher in East Asian countries than in Europe and in the United States. Blood As concentration was higher in male (10.85 μg/L) than female (10.02 μg/L) individuals (*p* = 0.018). Our data show a robust significant difference across age groups for blood As concentration; 13.52 μg/L for age group of 50–78 years versus 6.87 μg/L for the age group of 12–49 years (*p* < 0.001).

The GM of blood Cs levels is 2.39 μg/L in this study, which is similar in China (2.01 μg/L) [17] and a little less in India (1.0 μg/L) [18]. In the present study, GM of blood Cs levels was 2.65 μg/L for men and 2.45 μg/L for women (*p* < 0.001). A distinct pattern of age association in Cs could not be found. There is little information regarding the causes of increased Cs concentration in human blood with the exception of the two major nuclear accidents in Chernobyl and Fukushima.

In this study, the GM of blood Hg level was 3.41 μg/L and it is higher than other population. In 2011, Kim and Lee analyzed the data from the Korean National Health and Nutrition Examination Survey (KNHANES), conducted in 2005, and reported that the GM of the blood Hg level for 1997 Korean adult individuals was 4.15 μg/L [9]. This value is 4 to 5 times higher than that of Northern France (1.38 μg/L) [15], of the American population (0.83 μg/L) or of the Czech population in Europe (0.78 μg/L). Blood Hg level is significantly higher in Korean than in European populations, even compared to European countries in which a higher amount of fish is traditionally consumed, such as Sweden [19] or Italy [20]. These results could be explained by the greater seafood consumption among Asian populations compared to Europeans. Hightower et al. reported that Asians had the highest prevalence of elevated blood Hg among all racial groups [21].

The blood Hg concentration was significantly higher for male (5.92 μg/L) than female (3.31 μg/L) individuals (*p* < 0.01) in this study. A previous study, reported similar Hg concentration in the general adult population of 3.97 μg/L for men and of 2.62 μg/L for women [10]. This concentration difference between male and female individuals is due to differences in lifestyle, geographical differences concerning local Hg sources, and dietary habits. A distinct pattern of age-associated increase or decrease in Hg could not be found.

The GM of blood Al was 10.57 μg/L, with no significant difference between men and women and no age dependence. A previous study reported that the GM of blood Al was 8.61 μg/L for college students in Korea [22]. However, blood Al levels measured here, were considerably higher than those reported in France (2.32 μg/L) [15]. Al is one of the most abundant elements in the earth’s crust and is considered to be potentially toxic to humans and animals. Human diseases due to exposure to highly concentrated Al include aluminosis of the lung, Al encephalopathy, osteomalacia, myelotoxicity, and anemia. Dietary sources with the highest levels of Al are herbs and tea-leaves. Moreover, exposure to Al can also occur through water, food additives, and contamination during cooking or food storage (30–50 mg per day) [23]. The speculation that Koreans consume high amounts of herbs and tea cannot fully explain the high Al concentrations reported in the Korean population.

The GM of blood Cd in the Korean general population in this study was 0.78 μg/L which is relately similar to the Chinese population in Beijing (0.68 μg/L) [24], but higher than that reported for Germans (0.44 μg/L) [25], Italians (0.53 μg/L) [26]; and Brazilians (0.4 μg/L) [5]. Centers for Disease Control and Prevention (CDC) in the United States reported that Cd concentration in blood increases with age [26] in agreement with that our results showed that blood Cd was 120% higher for the group of 50–78 years than for the younger group of 12–49 years. Forte et al. also confirmed the age-related increase in blood Cd and pointed out that a plateau level can be reached in individuals above 50 years old, probably due to an age-related deterioration of kidney function [6,27]. Moreover, female individuals had higher blood Cd levels than males in the present study (1.00 μg/L vs. 0.83 μg/L), consistent with previous studies [6,28,29]. Generally, Cd retention is higher in women than in men [29]. The gastrointestinal absorption of dietary Cd is about 5% in adult men and 10% or higher in women [30,31,32]. However, individual values vary and are affected by factors such as dietary intake of essential nutrients (Fe, Ca, Zn and Cu) and protein. It has also been observed that Cd absorption may be increased in individuals with Fe deficiency [28], and partly explains the higher absorption of Cd by women [30]. Consequently, the difference in blood Cd becomes less obvious after menopause, when the Fe status in women improves [33].

Cu is an essential nutrient that is required for numerous metalloenzymes in various biochemical reactions in the body [34]. The GM of whole blood Cu in this study (980 μg/L) is slightly higher than previously reported values for Koreans (910 μg/L), Chinese (800 μg/L), Czech (800 μg/L), and Brazilian (890 μg/L) populations. However, our values are lower than the values reported for a German population (1020 μg/L) and for residents of Badajoz in Spain (1070 μg/L) [25,35]. GM of blood Cu tends to be 15–17% higher in women than in men based on previous literature reports [24,26,36]. The results of the current study are in line with previous literature data, showing about 13% higher Cu levels in women than in men. It has been hypothesized that estrogen-induced ceruloplasmin synthesis in the liver may lead to an increased Cu concentration in the blood [37]. In fact, blood Cu levels for 10-year-old girls and boys are nearly equal [38]. Bárány et al. also reported that there is no significant difference in blood Cu levels for 15-year-old boys and girls; but levels of blood Cu increase significantly for girls within the 2-year period from 15 to 17 years of age [39]. Consequently, 17-year-old girls have significantly higher blood Cu levels than boys of the same age [39]. Moreover, estrogens are known to directly influence copper metabolism, especially for girls that use contraceptives, contributing to increased plasma levels of this metal. Estrogen effects on copper levels are more evident in pregnant women, whose copper levels are higher compare to non-pregnant women [40,41,42]. The age of individuals did not affect blood Cu levels in our study, in line with the reports for an Italian population [26]. However, there are other reports that found an association of blood Cu levels with age [5,43].

Our data showed a GM of blood Mn of about 11.1 μg/L, which is similar to the value of 10.8 μg/L measured at the Korean national survey [10], and slightly higher than those reported for an Italian (8.9 μg/L) and a Brazilian (9.6 μg/L) population [5,26]. Our observation that women have a ∼11% higher GM of blood Mn than men, is consistent with the Korean national survey (11.7 μg/L in female vs. 9.9 μg/L in male individuals) [10] and with other literature reports. For example, there is a 20% higher GM of blood Mn for Chinese and Italian female population than their respective male population [24,26]. In addition, our data showed a slightly higher Mn in the young age group of 12–49 years (12.14 μg/L) than the one for the age group of 50–78 years (11.21 μg/L) (*p* = 0.05), in accordance with other reports [10,24].

In the present study, the mean serum Se level was 111.37 μg/L (SD = 22.32) which is similar to the level reported for other Asian countries such as Taiwan (110.9 μg/L) [44] and Japan (117.4 μg/L) [45], as well as Canada (115 μg/L) [46]. However, our Se values are generally lower than those obtained in the US during the National Health and Nutrition Examination Surveys (NHANES) of 2003–2004 (136.7 μg/L) [47]. In contrast, our serum Se concentration is higher than those reported in China (75.01 μg/L) [48], in the UK (women 79.7 μg/L; men 82.9 μg/L) [49], in Brazil (73.18 μg/L) [50] and in Spain (74.7 μg/L) [51]. In essence, Se concentrations in the populations of different countries differ considerably [48,49,50,51,52,53,54,55,56,57,58,59,60]. This variation can be attributed to different Se content in the soil of each country [61]. Moreover, we should consider that values measured can be influenced by the analytical method and the type of specimen used to assess Se. In the present study, we found no correlation between Se serum concentrations and age. However, other studies have reported positive or negative correlations [36,44,51]. These discrepancies observed regarding Se concentration correlation with age can be due to the different age groups selected in each study and differing diets [62]. In this study, men showed higher serum Se levels (117.45 μg/L) than women (110.06 μg/L), a result that is in agreement with previously reported data [63,64,65]. However, there are other studies that reported similar values for serum Se for both men and women [44,49,50].

In this study, the mean serum Zn concentration for the population was 872.7 µg/L (902.4 µg/L for male and 884.1 µg/L for female). Only a few studies have reported serum Zn concentration measured using ICP-MS in healthy Korean individuals. Previous studies had reported mean serum Zn concentrations of 804.5 µg/L [66] and of 1070 µg/L for 79 control individuals [67]. Zn levels reported in this study are higher than the ones reported for Brazilian populations (739 µg/L for male and 700 µg/L for female) [68], and for general Japanese women (606.9 µg/L) [69]. Our results did not show any significant differences in Zn concentration between men and women or between age cohorts.

## 5. Conclusions

This study has several strengths. First, this is the first study to assess 10 trace elements concentrations in the serum and blood within an ethnically homogeneous population of Korean. Second, the study sample size is enough to conduct a strong analysis. Finally, trace element concentrations were measured using the ICP-MS method by which multiple elements can be screened simultaneously and with high sensitivity.

This study also has a few caveats. First, it was a single referral laboratory study. Second, there were insufficient numbers of specimens for the age group below 30 years old. However, the current study is important because it is the first study to assess trace elements concentrations for a general healthy Korean population.

In conclusion, the element concentrations reported represent a new contribution to the knowledge of the blood chemistry for the Korean population. The data can be used to assess the clinical health of this population.

## Figures and Tables

**Table 1:** Age and gender of people who participated in this study.

Age, Years	No. of Subjects		
	Male	Female	Total
12–19	5	4	9
20–29	5	10	15
30–39	13	25	38
40–49	29	30	59
50–59	46	49	95
60–69	18	15	33
70–78	3	6	9
Total	119	139	258

**Table 2:** Blood element concentrations from people analyzed in this study.

Analyte ^a^	Specimen	GM ^b^	SD ^c^	Percentiles			
				2.5th	50th	95th	97.5th
Pb	Whole blood	15.97	8.312	7.40	16.20	34.22	36.37
As	Whole blood	7.19	10.018	1.27	6.95	34.15	40.34
Cs	Whole blood	2.39	1.161	1.30	2.40	3.92	4.26
Hg	Whole blood	3.41	3.861	0.60	3.30	11.32	14.72
Al	Whole blood	10.57	6.613	5.00	10.80	18.55	21.46
Cd	Whole blood	0.78	0.513	0.20	0.80	1.80	2.06
Cu	Serum	979.8	223.19	678.7	990.5	1370.3	1545.1
Mn	Whole blood	11.06	3.796	6.03	11.10	17.83	18.80
Se	Serum	111.37	22.320	79.07	110.60	154.75	166.46
Zn	Serum	872.7	202.16	555.2	871.5	1237.6	1287.1

^a^ Reported value in this study was μg/L; ^b^ GM, geometrical mean; **^c^** SD, standard deviation.

**Table 3:** Blood element concentration geometrical mean and age range analyzed in people < and ≥50 years.

Analyte	Age < 50 Years (*N* = 121)				Age ≥ 50 Years (*N* = 137)				*p* Value
	Min	Max	Mean	SD	Min	Max	Mean	SD	
Pb	1.2	58.1	16.59	8.552	1.8	42.6	19.35	8.802	0.011
As	0.8	35.0	6.87	5.709	0.6	59.8	13.52	11.834	0.000
Cs	1.2	4.6	2.46	0.710	0.3	16.0	2.61	1.452	0.296
Hg	0.3	19.6	4.05	3.269	0.4	25.9	4.92	3.893	0.054
Al	3.4	51.8	11.34	6.089	1.8	63.6	11.91	7.092	0.497
Cd	0.1	3.2	0.81	0.554	0.3	3.1	1.01	0.457	0.002
Cu	530	2508	1006.5	258.01	452	1588	998.2	189.02	0.765
Mn	5.6	27.8	12.14	3.858	4.5	24.7	11.21	3.714	0.050
Se	77.6	207.4	113.05	21.493	55.3	197.8	113.83	23.177	0.781
Zn	450	1689	897.5	185.01	531	2415	888.1	217.46	0.711

**Table 4:** Blood element concentration geometrical mean between genders.

Analyte	Male (*N* = 119)				Female (*N* = 139)				*p* Value
	Min	Max	Mean	SD	Min	Max	Mean	SD	
Pb	1.8	58.1	21.54	9.894	1.2	36.2	15.07	6.353	0.000
As	0.6	53.1	10.85	8.646	0.9	59.8	10.02	11.077	0.018
Cs	0.3	7.1	2.65	0.851	1.1	16.0	2.45	1.374	0.000
Hg	0.3	25.9	5.92	4.447	0.4	13.1	3.31	2.124	0.000
Al	3.4	63.6	11.62	7.734	1.8	56.2	11.66	5.550	0.147
Cd	0.2	3.2	0.83	0.463	0.1	3.1	1.00	0.543	0.004
Cu	452.0	1425	926.4	170.31	530.0	2508.0	1066.9	243.21	0.000
Mn	5.0	18.5	10.90	3.406	4.5	27.8	12.29	4.015	0.007
Se	55.3	207.4	117.45	24.629	70.4	171.7	110.06	19.680	0.009
Zn	450.0	1689.0	902.4	191.31	542.0	2415.0	884.1	212.04	0.131
